# Playing hide-and-seek with host macrophages through the use of mycobacterial cell envelope phthiocerol dimycocerosates and phenolic glycolipids

**DOI:** 10.3389/fcimb.2014.00173

**Published:** 2014-12-09

**Authors:** Ainhoa Arbues, GeanCarlo Lugo-Villarino, Olivier Neyrolles, Christophe Guilhot, Catherine Astarie-Dequeker

**Affiliations:** ^1^Centre National de la Recherche Scientifique, Institut de Pharmacologie et de Biologie StructuraleToulouse, France; ^2^Institut de Pharmacologie et de Biologie Structurale, Université de Toulouse, Université Paul SabatierToulouse, France

**Keywords:** mycobacteria, pathogens, virulence, lipids, macrophages, immune responses

## Abstract

Mycobacterial pathogens, including *Mycobacterium tuberculosis*, the etiological agent of tuberculosis (TB), have evolved a remarkable ability to evade the immune system in order to survive and to colonize the host. Among the most important evasion strategies is the capacity of these bacilli to parasitize host macrophages, since these are major effector cells against intracellular pathogens that can be used as long-term cellular reservoirs. Mycobacterial pathogens employ an array of virulence factors that manipulate macrophage function to survive and establish infection. Until recently, however, the role of mycobacterial cell envelope lipids as virulence factors in macrophage subversion has remained elusive. Here, we will address exclusively the proposed role for phthiocerol dimycocerosates (DIM) in the modulation of the resident macrophage response and that of phenolic glycolipids (PGL) in the regulation of the recruitment and phenotype of incoming macrophage precursors to the site of infection. We will provide a unique perspective of potential additional functions for these lipids, and highlight obstacles and opportunities to further understand their role in the pathogenesis of TB and other mycobacterial diseases.

## Introduction

Research progress has identified key players for mycobacterial pathogenicity including the major lipid virulence factor, phthiocerol dimycocerosates (DIM), and its structurally-related compound, phenolic glycolipids (PGL). In an elegant study, Cambier et al. proposed an exciting mechanism for their role in immune evasion strategies evolved by *Mycobacterium tuberculosis* (*Mtb*), and its close pathogenic relative *M. marinum* (Cambier et al., 2014). The objective of this perspective is to present a brief view of the literature concerning the immunomodulatory functions of DIM and PGL at the initial site of infection, and to propose a scenario for their molecular mechanism of action. In particular, we will address their impact on resident macrophages and recruitment of incoming precursors to the site of infection. Considering that very little is known about the actual interaction of these lipid virulence factors with the host immune system, a better understanding about their role in immune evasion may contribute to novel therapeutic strategies for mycobacterial diseases, such as tuberculosis (TB).

**Figure 1:**
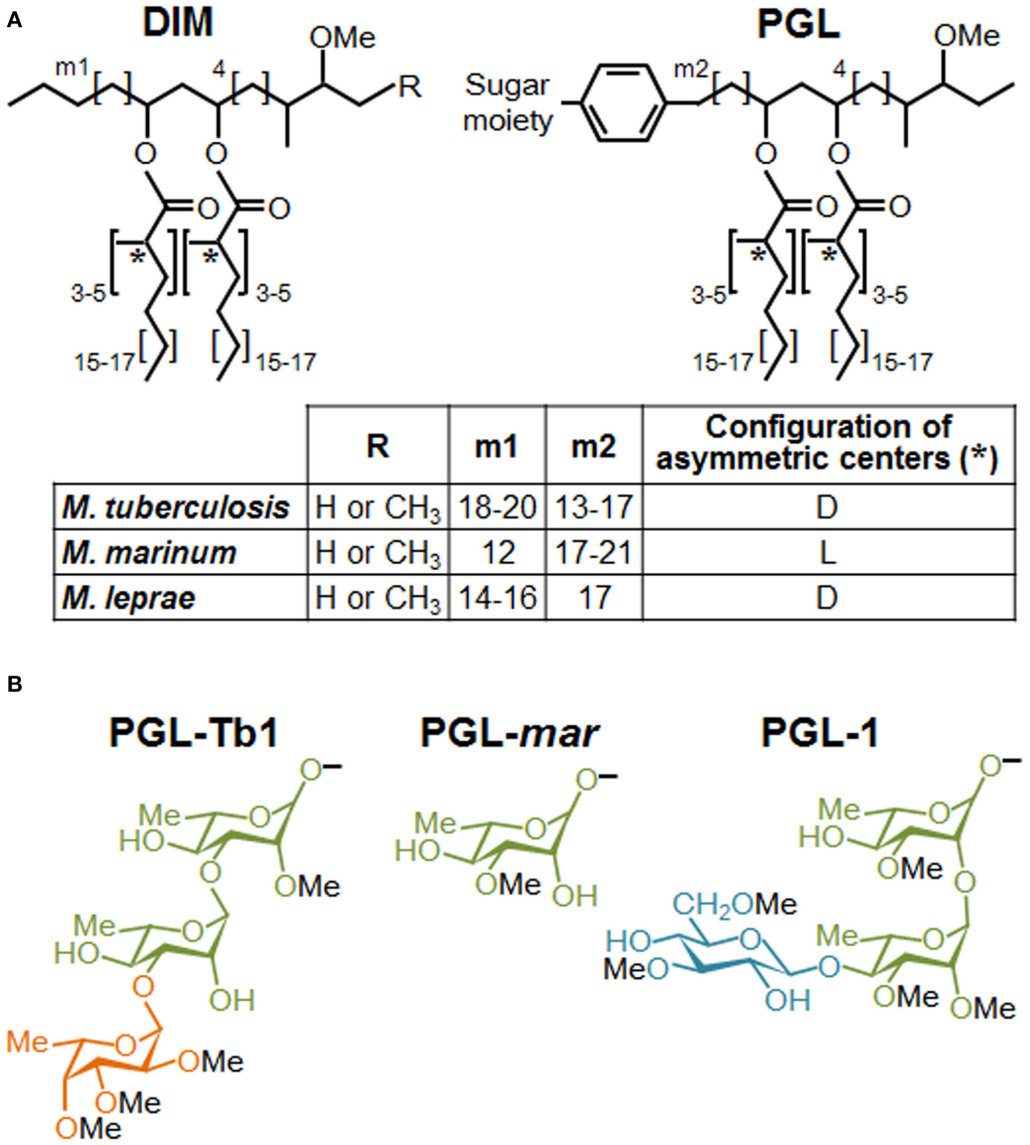
**Structures of DIM and PGL produced by various mycobacterial species. (A)** Structure of the major DIM and PGL lipid moieties. The lipid core is composed of a long-chain β-diol (phthiocerol and phenolphthiocerol), showing slight length variations (see m1 and m2 values in embedded table), esterified by polymethyl-branched fatty acids. In most cases, the configuration of the asymmetric centers bearing the methyl branches (asterisks) are of the D series, mycocerosic acids, but in a limited number of mycobacterial species, they belong to the L series and are then called phthioceranic acids (see table). Minor structural variants of the β-diol can contain a keto group in place of the methoxy group. **(B)** Structure of the species-specific sugar moiety of the major forms of PGL produced by *Mtb* (PGL-Tb1), *M. marinum* (PGL-*mar*) and *M. leprae* (PGL-1). Rhamnose is represented in green, fucose in orange and glucose in blue.

The virulence of mycobacterial pathogens is a multifaceted process that grants the means to circumvent a dedicated immune response and to thrive in host cells, including macrophages. In spite of recent progress demonstrating the contribution of mycobacterial cell envelope lipids to pathogenicity (for review see Guenin-Mace et al., 2009; Neyrolles and Guilhot, 2011), relatively little is known about their mechanism of action, and more specifically, how these lipids interact with the host at the molecular level. Most biological effects caused by lipids loosely associated to mycobacterial envelope derived from specific interactions with pattern recognition receptors (PRRs) on innate immune cells. Among the different PRR families, carbohydrate-recognition receptors (e.g., calcium-dependent (C)-type lectin receptors, CLR) have generated great interest because they recognize specific sugar moieties on glycolipids, referred to as PAMPs (pathogen associated molecular patterns). This, in turn, can trigger the internalization of mycobacteria and eventually a downstream signaling cascade that can generate opposite effects on host immune responses. For example, the macrophage inducible CLR Mincle recognizes the mycobacterial trehalose-6,6 dimycocerosate (TDM), likely through its trehalose motif, and triggers a pro-inflammatory cytokine production and Th1 and Th17 cell responses (Ishikawa et al., 2009; Schoenen et al., 2010). By contrast, the mannosylated moieties from the mycobacterial lipoglycans, ManLAM, mediate entry of the bacillus into macrophages through mannose receptor and DC-SIGN, whose signaling cascades engage an anti-inflammatory effect that enables *Mtb* to evade immune surveillance (for review see Mishra et al., 2011). Interestingly, the critical motifs of ManLAM for its recognition by DC-SIGN have been shown to be the mannose caps as well as the fatty acids (Maeda et al., 2003; Riviere et al., 2004). Moreover, data indicated that fatty acids are involved in a supramolecular organization of ManLAM, associated with an increased avidity for their receptors (Riviere et al., 2004). These findings strongly suggest that the lipid moiety of glycolipids interferes with their macromolecular organization, which may be necessary for efficient recognition of the terminal mannosyl epitopes. Lipids may also physically interfere with host membranes and thereby impair immune response-related signaling pathways, as proposed by Laneelle and Daffe (1991). Indeed, insertion of TDM in model or natural membranes can affect their biophysical properties (Laneelle and Tocanne, 1980; Sut et al., 1990; Almog and Mannella, 1996), decreasing notably membrane fluidity by ordering the surrounding phospholipids and cross-linking the leaflets (Laneelle and Tocanne, 1980; Almog and Mannella, 1996). Taken together, these data highlight the importance to consider the specific structural features of lipids in exploring their functional role. This is especially true for the highly hydrophobic DIM, and even more essential for their glycosylated version, PGL. DIM and PGL exhibit a common lipid backbone which is well conserved, with minor structural variations, among the few mycobacterial species synthesizing these molecules (Figure 1). In contrast, the saccharide domain of PGL is species-specific (Figure 1). Of note, all the major mycobacterial pathogens produce both substances, but in the particular case of *Mtb* only a subset of clinical isolates belonging to Beijing family is capable to synthesize PGL.

## Now you see me, now you don't: the hiding effect of DIM and more

The role of DIM in virulence was first suggested by two independent studies using signature-tagged transposon mutagenesis (Camacho et al., 1999; Cox et al., 1999), and thereafter supported by a large body of evidence (for review, see Neyrolles and Guilhot, 2011). Historically, the only well-established function for DIM was their structural support to the mycobacterial cell wall as protective permeability barrier (Camacho et al., 2001). For example, DIM deficient mutants are sensitive to reactive nitrogen species (RNS) generated in murine macrophages pretreated with IFN-γ and TNF-α (Rousseau et al., 2004). However, it is now apparent that other roles exist for DIM beyond the physical protection against host microbicidal factors, as mutants incapable of transporting DIM or spontaneous DIM deficient variants are more attenuated in mice lacking inducible nitric oxide synthase (iNOS) compared to control counterparts (Murry et al., 2009; Kirksey et al., 2011). Indeed, recent progress indicate that DIM play a dominant role in the modulation of protective host immune responses, specifically during the early steps of infection, when the bacilli encounter host macrophages (Rousseau et al., 2004; Astarie-Dequeker et al., 2009; Cambier et al., 2014; Passemar et al., 2014).

Two different but likely complementary mechanisms have emerged from the latest set of studies addressing this subject. On the one hand, Cambier et al. postulated that DIM mask physically mycobacterial PAMPs, and thus prevent their recognition by toll-like receptors (TLR) and the subsequent recruitment of microbicidal macrophages (Cambier et al., 2014). Using zebrafish to model *in vivo* the earliest interactions between bacteria and host macrophages, these authors reported that a *M. marinum* mutant lacking DIM on its surface was unable to prevent the recruitment of iNOS-expressing macrophages, which were effective in killing this mutant through RNS production. Further analysis indicated that DIM act by preventing TLR signaling via the common TLR adaptor MyD88 (Cambier et al., 2014). Supporting this notion, Rousseau et al. reported that infection of murine macrophages with a DIM-deficient *Mtb* mutant induced a high secretion of TNF-α and IL-6, two pro-inflammatory cytokines produced downstream of TLR- and MyD88-dependent signaling pathway (Rousseau et al., 2004). The authors also mentioned that the greater amount of TNF-α and IL-6 at the site of infection could overstimulate the recruitment of macrophages. The exact mechanism of how DIM are able to mask the mycobacterial PAMPs, and which TLR-containing immune cell is involved, remain to be described. On the other hand, our group proposed that DIM target lipid organization in the membrane of host macrophages, thereby modifying its biophysical properties and subsequently the activity of membrane effectors (Astarie-Dequeker et al., 2009). We observed that DIM-deficient mutants are poorly efficient to infect human macrophages, and accumulate in acidified phagosomes at early time post-infection. Importantly, prevention of phagosomal acidification by the proton-ATPase inhibitor, bafilomycine, rescued the growth defect of a DIM deficient mutant (Passemar et al., 2014). This suggests that DIM contribute to the intracellular growth of *Mtb* by excluding somehow the proton-ATPase from the phagosomal membrane. At late post-infection stages, DIM-deficiency affects the capacity of *Mtb* to induce cell death and to disseminate into bystander macrophages (Passemar et al., 2014). While these results should be interpreted with caution, they suggest that DIM participate in control of the outcome of infected macrophages and cell-to-cell spread of *Mtb*. At the root of these observations, we noticed that *Mtb* interacts with macrophages in a manner that induces changes in membrane fluidity along with the requirement of DIM at the bacterial surface (Astarie-Dequeker et al., 2009). The ability of DIM to induce changes in membrane fluidity is consistent with their hydrophobic properties that would facilitate their insertion into membranes. As non-covalently bound molecules to the outer cell wall layer of mycobacteria, DIM could thus insert into membranes of macrophages to induce biophysical changes that would modulate the activity of membrane-associated effectors. At the level of the plasma membrane, this would increase macrophage infection by enhancing the activity of phagocytic receptors (e.g., complement receptor 3, CR3), and subsequent bacterial replication through inhibition of phagosome acidification (Astarie-Dequeker et al., 2009; Passemar et al., 2014). Moreover, DIM could also collaborate with the secreted *Mtb* protein, ESAT-6 by modulating its membrane lytic activity. Indeed, DIM and ESAT-6 share the capacity to induce cell death and cell-to-cell spread of *Mtb* (Aguilo et al., 2013; Passemar et al., 2014), two events associated to membrane damages.

Collectively, these results clearly established that DIM play an active role at modulating the recognition of mycobacterial PAMPs and avoiding the activation of pro-inflammatory macrophages, and at the same time, altering macrophage responses to its favor possibly by provoking changes in the membrane fluidity that ultimately affect the microbicidal functions.

## Seeking the right partner to play: the significance of PGL structural variability

PGL possess a common lipid core closely related to DIM that, consequently, may share the properties just described for these molecules. However, PGL also exhibit species-specific saccharide domains (Figure 1) and this variation in sugar content may have a strong impact on the PRRs involved in their recognition, and therefore, on their biological activities.

Most of *Mtb* clinical isolates, as well as the reference laboratory strains, are unable to produce PGL (Constant et al., 2002). Yet, some *Mtb* strains are able to synthesize a specific form of PGL named PGL-Tb1. This strongly suggests that PGL are not essential for TB pathogenesis. However, their presence may have an impact on the interaction with the host and increases *Mtb* virulence. Indeed, Reed et al. established that the production of PGL-Tb1 in a Beijing clinical isolate is associated with a hypervirulent phenotype in animal models (Reed et al., 2004). It was also reported that treatment of mouse macrophages with purified PGL-Tb1 inhibits the production of the pro-inflammatory cytokines TNF-α, IL-6 and CCL2, and that this effect was dependent on its saccharide domain. The activity of PGL-Tb1 on the modulation of the immune response was also supported by independent results showing that the production of this lipid in the laboratory strain H37Rv, usually unable to synthesize it, modifies cytokine secretion by infected macrophages (Sinsimer et al., 2008). However, in that series of experiments, PGL-Tb1-producing H37Rv was not found to be more virulent than the parental strain in mice. Pointing to the relevance of this molecule, PGL-deficient *Mtb* strains were found to release *p*-hydroxybenzoic acid derivatives (*p*-HBAD), a truncated form of PGL containing just the saccharide domain and the phenol ring, which may also have biological activities (Constant et al., 2002). Mutants unable to produce PGL-Tb1 glycosylated-phenol moiety (*p*-HBAD II) induced increased secretion of the pro-inflammatory cytokines TNF-α, IL-6 and IL-12p40 (Stadthagen et al., 2006).

PGL are also produced by the fish pathogen *M. marinum*, used in several laboratories to model host-pathogen interactions in the context of human TB. PGL-*mar* (Figure 1) was found to be a key factor in the phagosomal maturation arrest induced by *M. marinum* (Robinson et al., 2008). In addition, the ability of purified PGL-*mar* to abrogate the secretion of pro-inflammatory cytokines TNF-α and IL-12p40 by human macrophages was also described. Little more was known about the biological activities of this molecule until recently, when it was demonstrated that PGL-*mar* is involved in the chemokine (C-C motif) receptor 2 (CCR2)-dependent recruitment of macrophages by inducing the expression of the chemokine CCL2 through a molecular mechanism still unknown (Cambier et al., 2014). Indeed, by selectively recruiting pathogen-permissive macrophages, Cambier et al. demonstrated that PGL-*mar*, in a proposed coordinated manner with the masking effect of DIM, increases *M. marinum* fitness in the host.

Finally, PGL-1 from *M. leprae* has retained special attention among the different PGL species since its discovery in the early 1980s, mainly due to the high immunogenicity of its sugar moiety (for a review see Spencer and Brennan, 2011). Like PGL-Tb1, PGL-1 contains a trisaccharide domain but the structure is different (Figure 1). Mainly due to the inability to grow the leprosy bacillus *in vitro*, there is a large body of literature using isolated PGL-1 to study its role in the modulation of host immune (Mehra et al., 1984; Schlesinger et al., 1994). Among others, PGL-1 was found to inhibit the pro-inflammatory cytokine secretion by human monocytes (Silva et al., 1993). In addition, *M. leprae* induces a poor activation and maturation of dendritic cells, and dampens their ability to induce T-cell responses (Hashimoto et al., 2002). This inhibition was partially relieved by treatment of *M. leprae*-infected cells with anti-PGL-1 antibodies. Together, those studies suggest that PGL-1, through its terminal trisaccharide motif, is the factor responsible for the immunosuppression observed in lepromatous leprosy, one of the most characteristic forms of the disease. Using an original genetic engineering strategy, our team has demonstrated that PGL-1 expression in *M. bovis* BCG enables this recombinant strain to exploit CR3 to promote bacterial uptake and to inhibit TNF-α secretion by human macrophages (Tabouret et al., 2010). In line with this finding, a recent study showed that synthetic PGL-1 saccharide moiety reduces the production of cytokines (TNF-α, IL-6, IL-1β and CCL2) induced by TLR-2 (Elsaidi et al., 2013).

These results indicate that mycobacteria make use of PGL to modulate host innate immune response, and that this activity is primarily mediated by their saccharide domain through an unknown molecular mechanism. Our data also support the notion that some PGL forms, such as PGL-1, could enable mycobacteria to take advantage of CR3 (Tabouret et al., 2010) to invade macrophages and exert anti-inflammatory properties by interfering with the TLR-dependent pro-inflammatory responses, as previously described for other pathogens (Wang et al., 2007; Hajishengallis and Lambris, 2011; Dai et al., 2013). However, we cannot rule out the possibility of a direct inhibition of TLR-dependent cytokine secretion, or the direct interaction with a “PGL receptor” on epithelial cells, is involved in the induction of CCL2 and subsequent macrophage recruitment, as hypothesized by Cambier et al. for *M. marinum* (Cambier et al., 2014). Finally, it is important to keep in mind that the structure of the saccharide domain of PGL is highly variable and species-specific (Figure 1). Therefore, it remains to be seen whether the strategy involved in immunomodulation is the same or varies for each PGL variant, and whether a PGL variant is more active than others.

## Conclusion: playing hide-and-seek with host macrophages

From these data, it emerges that pathogenic mycobacteria utilize DIM, on their own or in conjunction with PGL (depending on mycobacterial strains) for diverting macrophages from their natural function and for establishing infection. Most of the research has focused on the macrophage because its dialog with mycobacteria is thought to be the seminal step of the immune response. The study of Cambier et al. marks an important step in understanding the functions of lipids, as it takes into account the full repertoire of the innate immune system (Cambier et al., 2014). However, several questions remain pending: for example, what is the population of recruited macrophages? If we refer to the model illustrating changes in macrophage polarization throughout the infectious process (Lugo-Villarino et al., 2011), lipids might mediate evasion of microbicidal functions of M1 macrophages in the early stage of infection, whereas they might favor recruitment of M2 macrophages with weak microbicidal competences later on during infection. It also remains to be determined how lipids behave within macrophages with distinct phenotype/polarization in order to assess whether, by virtue of their pleiotropic effect, they have an intracellular impact throughout the disease.

**Figure 2:**
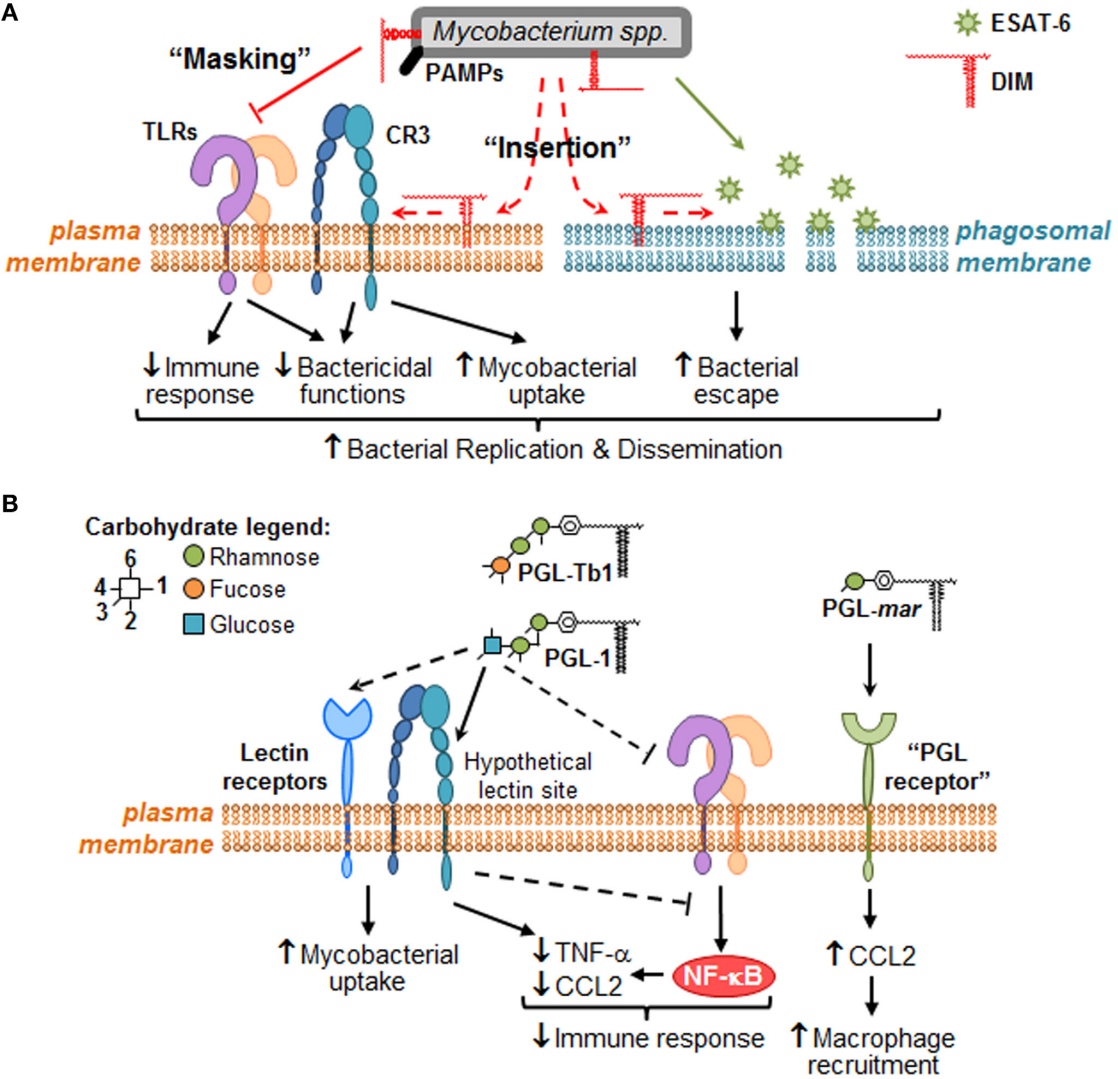
**Putative molecular mechanisms through which DIM and PGL remodel macrophage activity during the early steps of infection**. When mycobacteria encounter macrophages, they use cell surface exposed PAMPs to recognize numerous plasma membrane PRRs, such as TLR (e.g., TLR-2) and carbohydrate-recognition receptors (e.g., CR3 or other lectin receptors). **(A)** During bacterial recognition, DIM exert a masking effect on PAMPs, thereby preventing TLR-2 detection and triggering of subsequent bactericidal and immune responses. DIM could also insert into plasma membrane, changing its biophysical properties in a manner that increases CR3-mediated bacteria uptake, and decreases bactericidal functions. Likewise, DIM could insert within intracellular membranes (e.g., phagosomal membranes) where they collaborate with ESAT-6 to increase its membrane lytic activity, thereby inducing membrane damage and consequently allowing mycobacteria to escape into the cytosol. Altogether, DIM-mediated effects may lead to mycobacteria replication and innate immune evasion. **(B)** Some PGL species, such *M. leprae* PGL-1 and potentially *Mtb* PGL-Tb1, are able to decrease host immune response either by direct inhibition of TLRs or by taking advantage of lectin receptor (e.g., CR3) capacity to interfere with TLR-triggered pro-inflammatory cytokine secretion (e.g., TNF-α). They may also exploit these lectin receptors to promote mycobacterial uptake by macrophages. In the case of PGL-*mar*, a putative “PGL receptor” at the surface of epithelial cells has been proposed to be responsible for the induction of CCL2, and the subsequent macrophage recruitment.

We anticipate that the identification of the molecular mechanisms involved in *Mtb* lipid effects will lead to the characterization of signaling pathways that are modulated for the benefit or detriment of mycobacteria survival in the host. Therefore, we would like to provide a framework to study the molecular mechanisms governing mycobacterial DIM and PGL activity at the direct interface with macrophages (Figure 2). We propose that DIM exert more than a masking effect of cell wall exposed *Mtb* PAMPs (Cambier et al., 2014) and play an active role through their hydrophobic part to reshape macrophage activity (Figure 2A). We also propose that PGL, on their part, act through their saccharide domains as potential ligands for carbohydrate-recognizing receptors expressed at the surface of macrophages (Figure 2B).

For the purpose of conciseness, we focused the current perspective on DIM and PGL, but we believe that in the near future there will be a need for a broad assessment of mycobacterial lipids and their overall effects in the immune system. Novel strategies need to be devised in order to deal with the redundant role as virulence or immunomodulatory factor among mycobacteria cell envelope lipids. In this aspect, our group has undertaken the construction of single and multiple lipid mutants to assess the role of trehalose-derived lipids, sulfolipids, diacyltrehaloses and polyacyltrehaloses, and their respective contribution to the virulence together with DIM (Passemar et al., 2014). Similarly, there is a well-known redundancy among host PRRs that may hamper the characterization of signaling pathways triggered by mycobacterial lipids; our group has also made headways to develop techniques for the simultaneous double-gene silencing in primary mononuclear phagocytes (Troegeler et al., 2014). Finally, PGL variants merit careful consideration, as there is a strong potential for variation at the functional level according to species. This is particularly true for the assessment of permissive macrophage recruitment toward the site of infection for both PGL-Tb1 and PGL-1. Unlike the inducing effect by PGL-*mar* in the context of zebrafish infection (Cambier et al., 2014), both PGL-Tb1 and PGL-1 inhibit the CCL2 secretion by macrophages (Reed et al., 2004; Elsaidi et al., 2013). This divergence could be due to a differential effect of PGL depending on cellular context, or more interestingly, through a distinctive recognition by the involved receptor according to the saccharide composition. To address this type of question, the strategy developed by our team based on the use of *M. bovis* BCG as surrogate might be a valuable tool to enable the direct comparison of the effects of the PGL variants in the context of a relevant mycobacterial envelope and within the same genetic background (Tabouret et al., 2010).

In conclusion, we believe that the development of microbiological tools and adequate research models, in combination with multidisciplinary strategies, should open up new venues to achieve a better understanding of the ever-evolving relationship between host and pathogen. In many ways, the lipid at the mycobacteria wall is just a starting point!

### Conflict of interest statement

The authors declare that the research was conducted in the absence of any commercial or financial relationships that could be construed as a potential conflict of interest.
